# Supplementary material for: Sec14l3 potentiates VEGFR2 signaling to regulate zebrafish vasculogenesis
Source: Nat Commun. 2019 Apr 8;10:1606. doi: 10.1038/s41467-019-09604-0 (PMC6453981; doi:10.1038/s41467-019-09604-0)
Supplement: Supplementary file 1 — Supplementary Information [file 41467_2019_9604_MOESM1_ESM.pdf]

Sec14l3 potentiates VEGFR2 signaling to regulate zebrafish vasculogenesis

Gong et al.

Supplementary Figure 1, related to Figure 1 and Figure 2

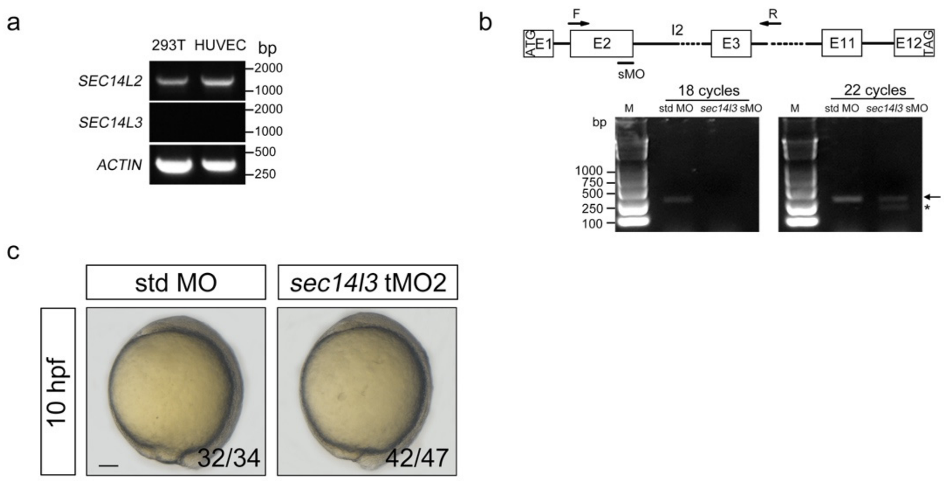

Supplementary Figure 1. ***SEC14L2*****/*****SEC14L3*** **mRNA expression in HEK293T and HUVEC cells, and the effectiveness of *sec14l3* sMO.** (**a**) Total mRNAs extracted from these two cell lines were used for generating cDNA libraries, and then RT-PCR of *SEC14L2* and *SEC14L3* was performed. *ACTIN* serves as a loading control. (**b**) the effectiveness of *sec14l3* sMO. The target site of *sec14l3* sMO is localized at the junction of the exon 2 and intron 2. Embryos injected with 0.5 ng std-MO or sec14l3-sMO were harvested for total mRNA extraction and cDNA preparation. PCR primers are outlined above the gene diagram as "F" and "R", and semi-quantitative PCR result shows that sec14l3-sMO injection results in the diminished transcript (indicated by the arrow) and production of an aberrantly spliced mRNA (donated by the star). (**c**) 5 ng sec14l3-tMO2 injection has no effect on embryonic CE movements at the gastrulation stage. Scale bars, 100 μm.

Supplementary Figure 2, related to Figure 2

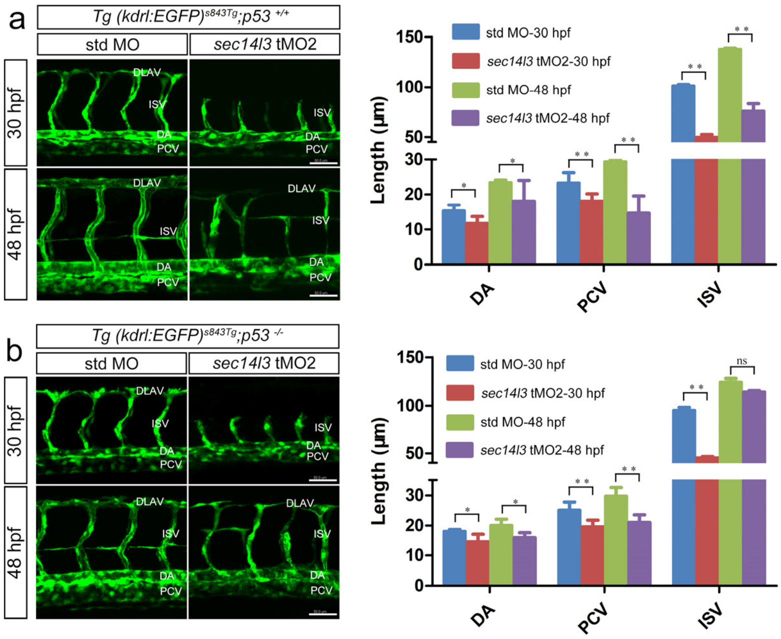

#

Supplementary Figure 2. *p53* mutation could partially rescue the angiogenic defects caused by *sec14l3* knockdown. (**a**) Vascular defects at 30 hpf and 48 hpf caused by sec14l3-tMO2 knockdown in *Tg(kdrl:GFP)*^{s843Tg}*;p53*$^{+/+}$ embryos. 5 ng sec14l3-tMO2 was injected at 1-cell stage embryos from *Tg(kdrl:GFP)*^{s843Tg}*;p53*$^{+/+}$ fishes and vascular defects were checked at 30 hpf and 48 hpf. Scale bars, 50 μm. The right histogram shows the statistical analyses of the luminal diameters of DA and PCV, as well as the length of ISV at 30 hpf and 48 hpf in (**a**). Data are shown as mean ± SEM (n=20 embryos) of each group. *, $p < 0.05$; **, $p < 0.01$. **(b)** Vascular defects at 30 hpf and 48 hpf caused by sec14l3-tMO2 knockdown in *Tg(kdrl:GFP)*^{s843Tg}*;p53*$^{-/-}$ embryos. 5 ng sec14l3-tMO2 was injected at 1-cell stage embryos from *Tg(kdrl:GFP)*^{s843Tg}*;p53*$^{-/-}$ fishes and vascular defects were checked at 30 hpf and 48 hpf. Scale bars, 50 μm. The right histogram shows the statistical analyses of the luminal diameters of DA and PCV, as well as the length of ISV at 30 hpf and 48 hpf in (**b**). Data are shown as mean ± SEM (n=20 embryos) of each group. *, $p < 0.05$; **, $p < 0.01$; ns, not significant.

Supplementary Figure 3, related to Figure 2

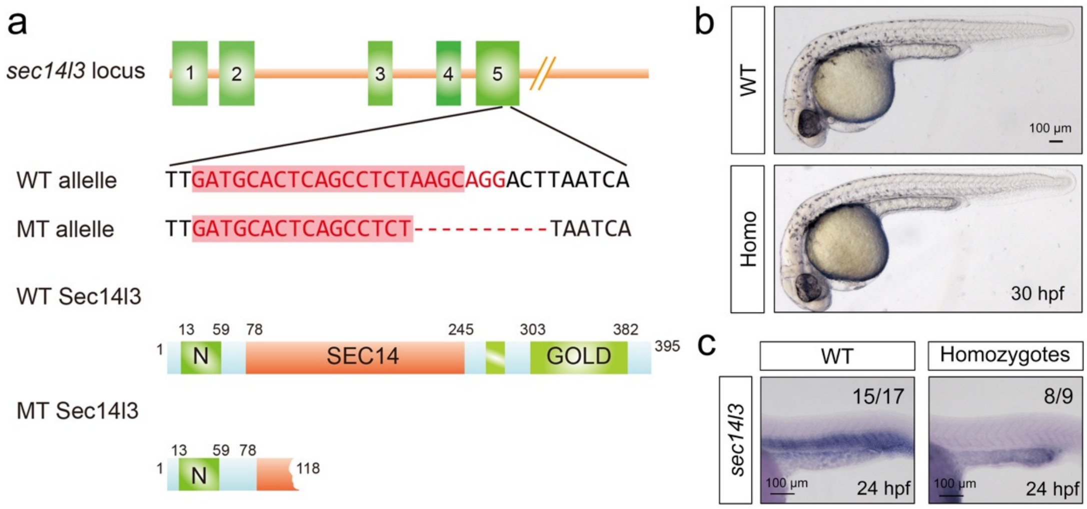

Supplementary Figure 3. Schematic of *sec14l3* mutant generated by CRISPR/Cas9 tool and *sec14l3* expression in the mutant. (**a**) Cartoon of *sec14l3* mutant information. gRNA is designed at the 5th exon and its sequence is shown in red with shade. A mutant line with 10 bp deletion is also shown, resulting in truncated Sec14l3 protein, missing SEC14 and GOLD domains. (**b**) Morphology of WT and homozygotes at 30 hpf. (**c**) Expression of *sec14l3* in WT and homozygotes at 24 hpf. Embryos from heterozygotes intercrossing were harvested for WISH and genotyped individually after staining.

Supplementary Figure 4, related to Figure 2

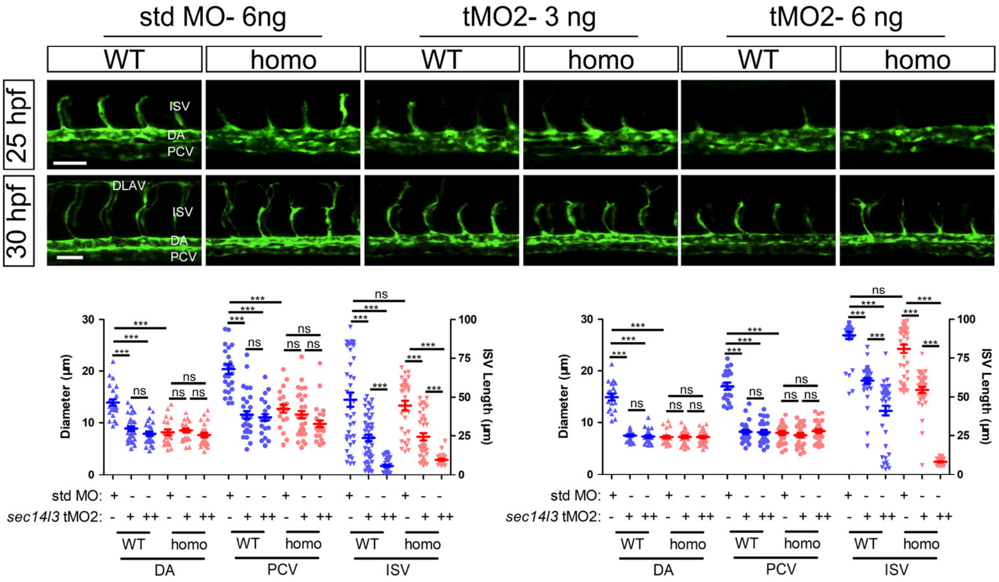

Supplementary Figure 4. sec14l3-tMO2 injection has no effect on DA and PCV diameters in *sec14l3*$^{-/-}$ mutants. 1-cell stage embryos from intercrossing *sec14l3*$^{+/-}$ heterozygotes in *Tg(kdrl:GFP)*^{s843Tg} background were harvested for injection with 6 ng std-MO or 3 ng / 6 ng sec14l3-tMO2. At 25 hpf and 30 hpf, DA/PCV luminal diameters and ISV length were checked for each embryo, which was lysed for genotyping analysis and grouped according to its genotype. Vascular morphology of each group is shown in the upper panel, while the statistic results of the DA/PCV luminal diameters and ISV length are shown in the bottom panel. To quantify the DA and PCV luminal diameters for an embryo, five different vessel regions along the yolk extension site of the same embryo were measured and their average value was used to represent its actual vessel diameter. As for ISV length, ten ISVs above the yolk extension region were measured for average value calculation to represent the final ISV length. Three independent experiments were carried out and data were shown as mean ± SEM. At 25 hpf, the numbers of observed embryos from left to right are 25, 30, 25, 30, 30 and 27 respectively; at 30 hpf, the numbers of observed embryos from left to right are 20, 25, 24, 25, 25 and 30 respectively. ***, $p < 0.001$; ns, not significant. Source data are provided as a Source Data file.

Supplementary Figure 5, related to Figure 2

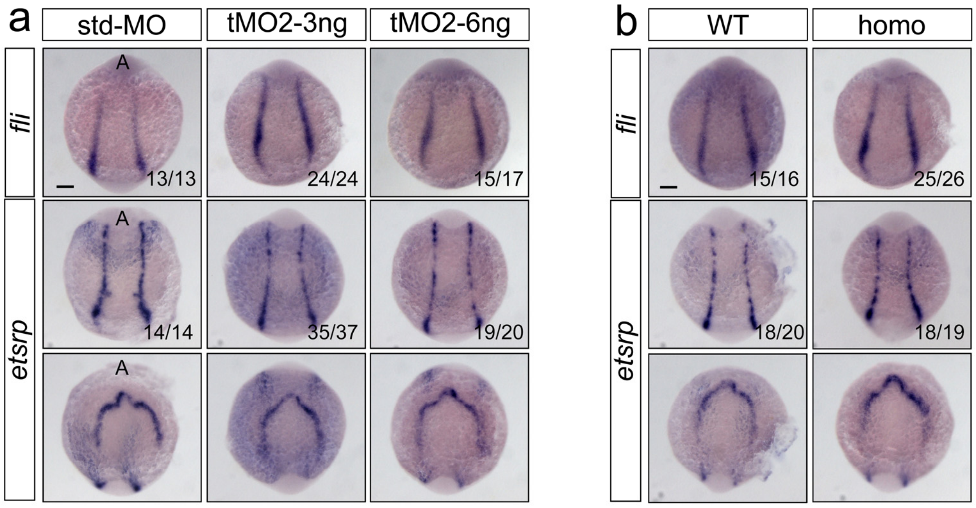

Supplementary Figure 5. Knocking down *sec14l3* has no effect on angioblast specification at 8-somite stage. (a) sec14l3-tMO mediated knockdown of *sec14l3* has no effect on *fli* or *etsrp* expression at 8-somite stage. 6 ng std MO or 3 ng / 6 ng sec14l3-tMO2 was injected into 1-cell stage embryos. *fli* and *etsrp* probes are used to indicate the trunk angioblasts. The ratio in the right corner indicates the number of embryos with altered expression pattern/the number of observed embryos. Embryos were dorsally viewed with anterior to the top. Scale bars, 100 μm. **(b)** *fli* or *etsrp* expression in *sec14l3*$^{-/-}$ homozygotes. *sec14l3*$^{+/-}$ heterozygotes were intercrossed for embryos harvest at the 8-somite stage, and after WISH with *fli* and *etsrp* probes, embryos were lysed individually for their genotyping analysis and grouped according to their genotypes.

Supplementary Figure 6, related to Figure 2

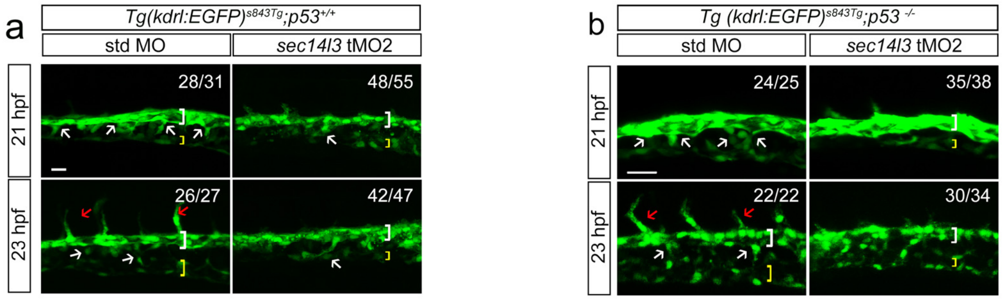

Supplementary Figure 6. sec14l3-tMO mediated knockdown of *sec14l3* impairs ventral sprouting of venous progenitors independent of *p53*. 5 ng sec14l3-tMO2 was injected into 1-cell stage embryos of *Tg(kdrl:GFP)*^{s843Tg}*;p53*$^{+/+}$(**a**) or *Tg(kdrl:GFP)*^{s843Tg}*;p53*$^{-/-}$ (**b**) fish, and then the trunk arterial-venous angioblast sorting and segregation were checked at indicated stages. White and yellow brackets indicate DA and PCV respectively; white and red arrows indicate venous endothelial progenitor cells sprouting from vascular cord and ISV sprouting from DA respectively. Scale bars, 25 μm.

Supplementary Figure 7, related to Figure 3

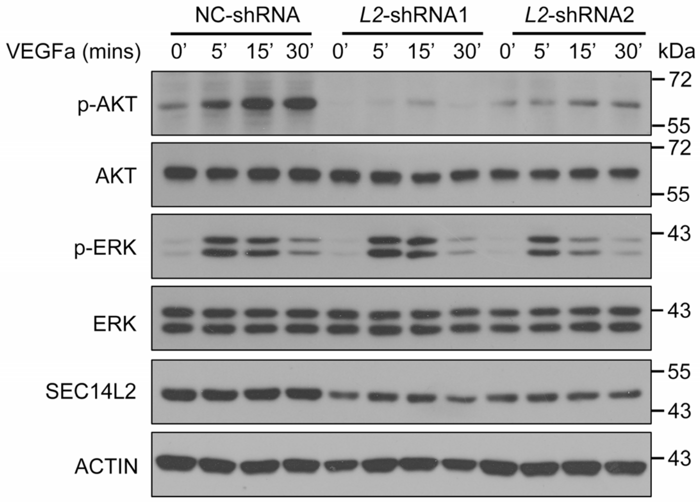

Supplementary Figure 7. *SEC14L2* knockdown mainly counteracts VEGFa-motivated p-AKT level in HUAECs. HUAECs were infected with NC or *SEC14L2* shRNA for 48 h and starved overnight before addition of 100 ng/ml VEGFa. After VEGFa stimulation for 5, 15 or 30 min, cell lysates were harvested and immunoblotted with indicated antibodies.

Supplementary Figure 8, related to Figure 5

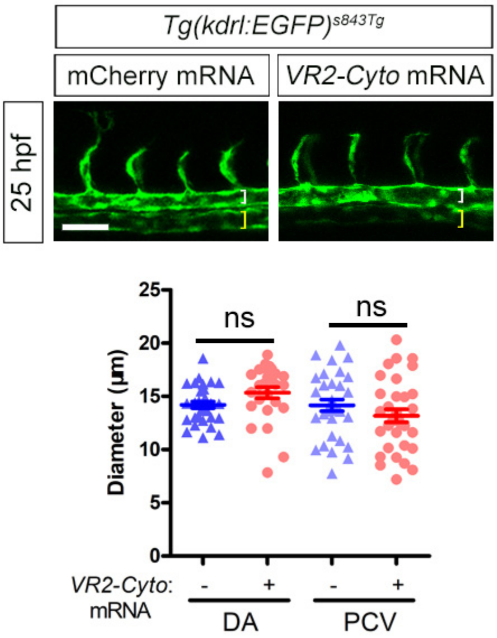

Supplementary Figure 8. *VEGFR2-cyto* mRNA overexpression has no significant effect on the vasculature in zebrafish at 25 hpf. 50 pg *VEGFR2-cyto* mRNA was injected into embryos from *Tg(kdrl:EGFP)*^{s843Tg} transgenic fish. Embryos were harvested at 25 hpf for vasculature observation. The lower panel shows the statistic results of DA and PCV diameters as mean ± SEM (n=30 embryos). ns, not significant. Scale bar: 50 µm.

Supplementary Figure 9, related to Figure 5

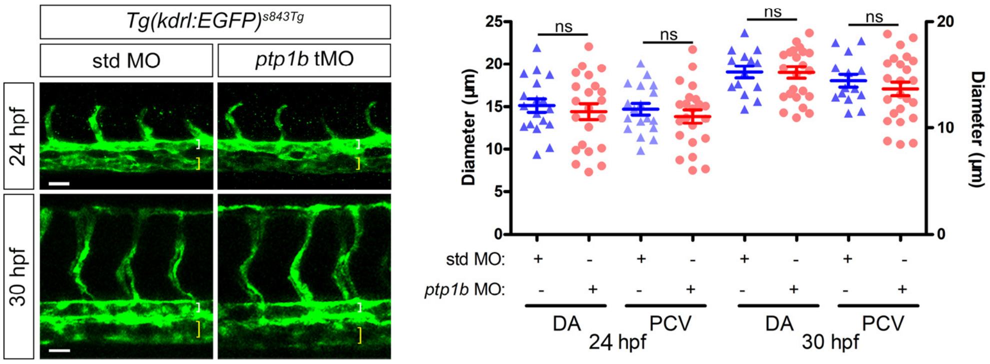

Supplementary Figure 9. *ptp1b* knockdown has no obvious effect on the vasculature in zebrafish at 24 hpf and 30 hpf. 5 ng *ptp1b*-tMO was injected into embryos in a *Tg(kdrl:EGFP)*^{s843Tg} transgenic background. At 24 hpf and 30 hpf, embryos were harvested respectively for vasculature examination. The right panel shows the statistic result of the luminal diameters of DA and PCV from three independent experiments. 20 embryos were calculated for each group and data are shown as mean ± SEM. ns, not significant. Scale bars: 25 μm

Supplementary Figure 10, related to Figure 6

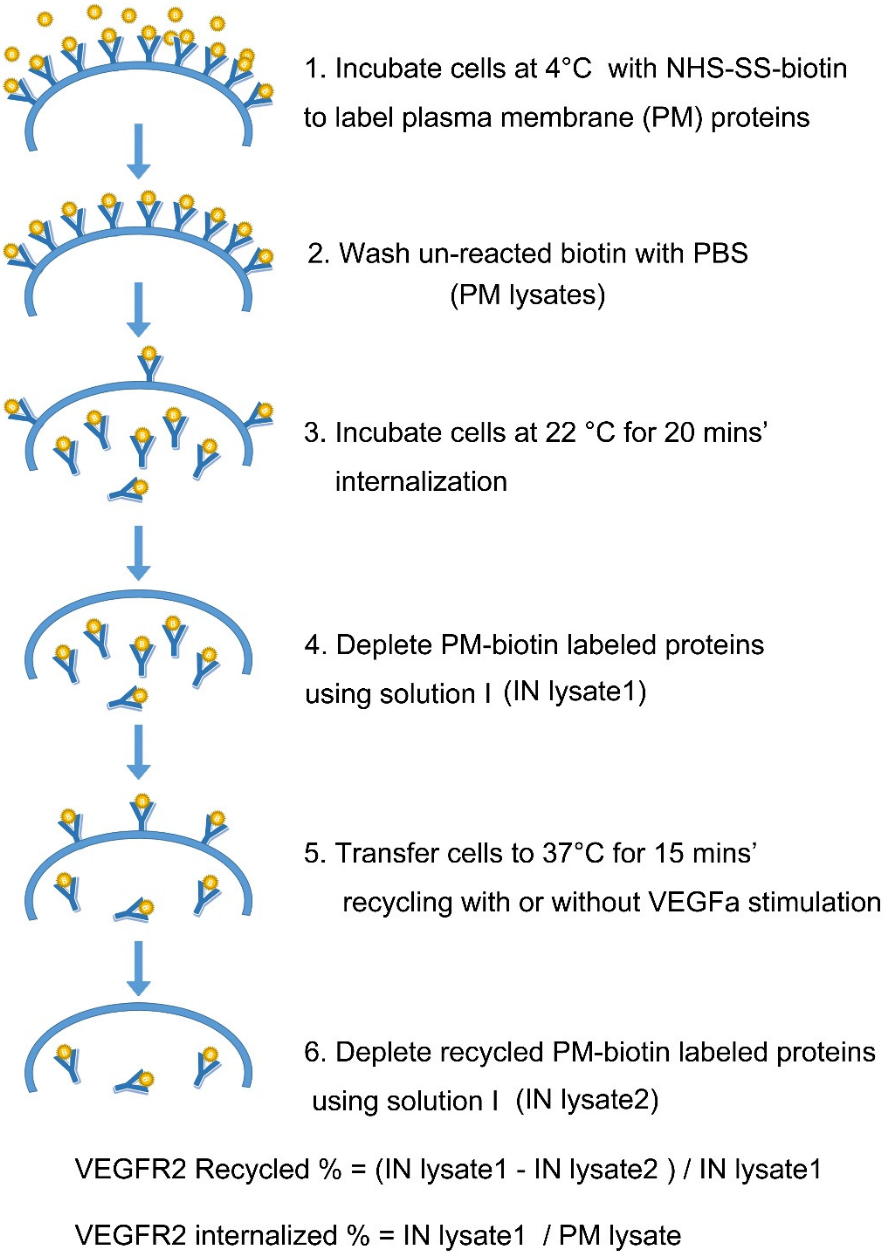

Supplementary Figure 10. Cartoon protocols of internalization and recycling assay based on NHS-SS-Biotin labeling. The schematic mainly illustrates the procedure of recycling assay, with six steps above followed by cell lysate harvest and streptavidin-beads incubation to pull down biotin (yellow balls) labeled proteins (blue “Y” shape). For the internalization assay, only the first four steps are involved with a slight modification in the third step that internalization initiates at 37°C for 5 min or 10 min.

Supplementary Figure 11, related to Figure 6

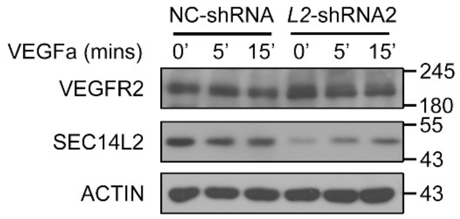

Supplementary Figure 11. *SEC14L2* knockdown has no effect on VEGFR2 protein level. Western blot analysis of cell lysates from *NC* or *SEC14L2* shRNA infected HUVECs. Cells were infected and starved overnight, followed by 100 ng/ml VEGFa stimulation for 5 or 15 min respectively. VEGFR2, SEC14L2 and ACTIN protein levels were examined by immunoblotting using respective antibodies.

Supplementary Figure 12, related to Figure 8

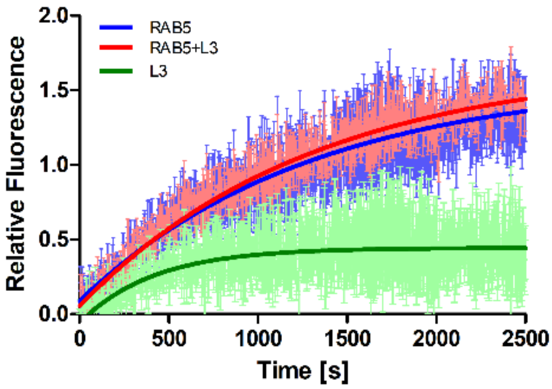

Supplementary Figure 12. Sec14l3 could not accelerate the GTP loading activity of RAB5. Sec14l3 protein and RAB5 protein were individually expressed and purified for the exchange assay based on the fluorescence of Mant-GTPγS. The time-course fluorescence was measured at 20℃ (λ_{ex}=355 nm and λ_{em}=488 nm), following the addition of 2 μM RAB5, 0.8 μM Sec14l3 or the mixture of 2 μM RAB5 and 0.8 μM Sec14l3 protein. Three independent experiments were performed and here shows one representative data of GTP loading curves. Different colors indicate distinct groups, RAB5 alone in blue, Sec14l3 alone in green and their mixture in red.

Supplementary Figure 13, related to Figure 3-8

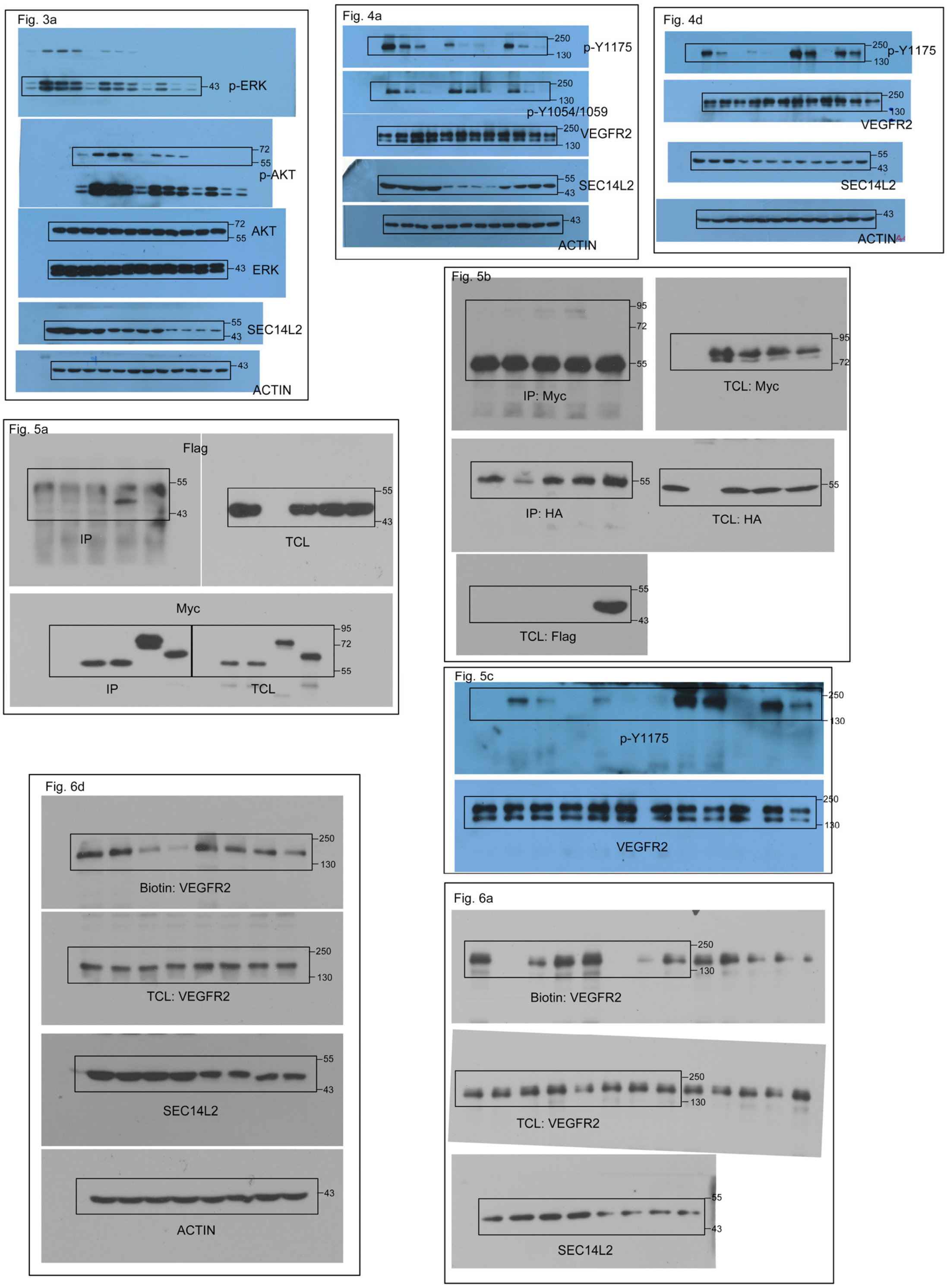

Fig. 7a

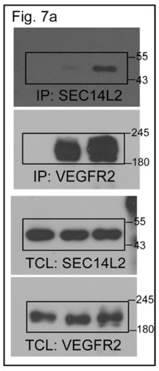

Fig.7b

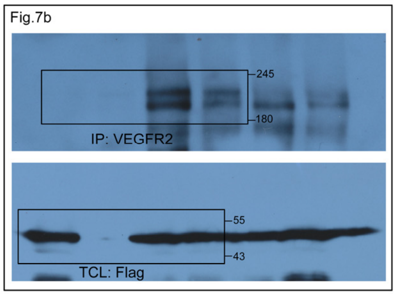

Fig.7c

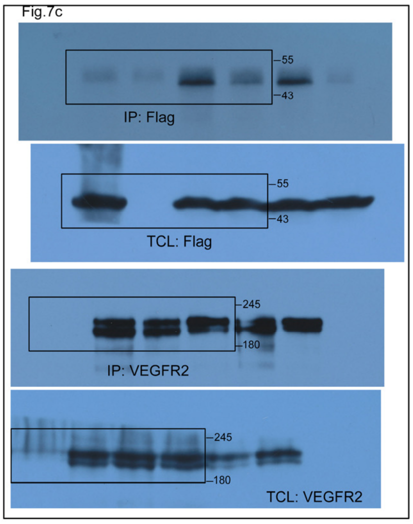

Fig.7e

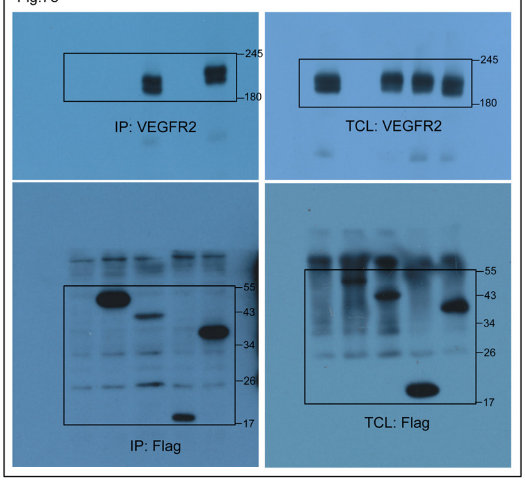

Fig.7f

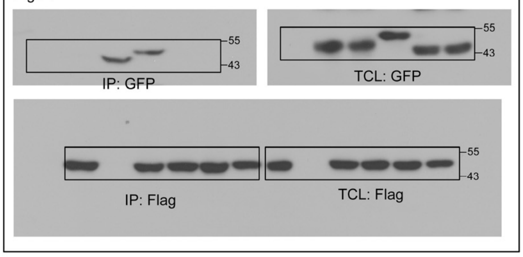

Fig.7i

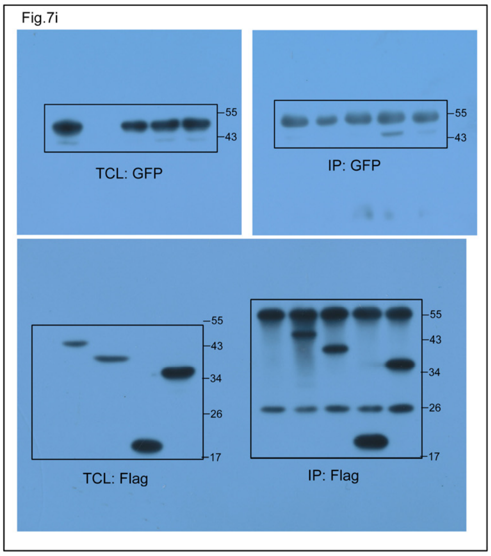

Fig.7g

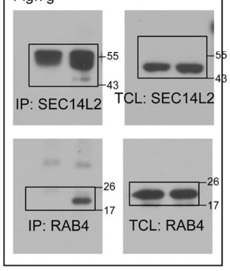

Fig. 7h

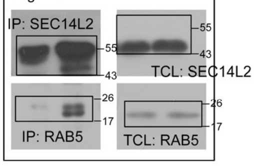

Fig. 8a

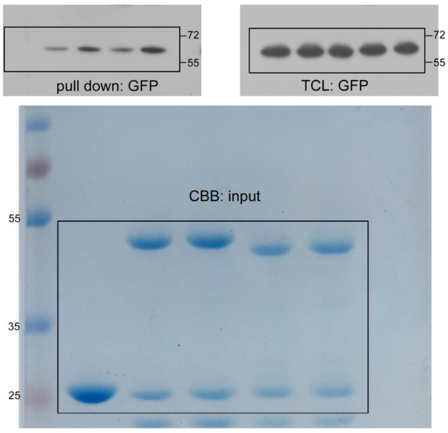

Fig. 8c

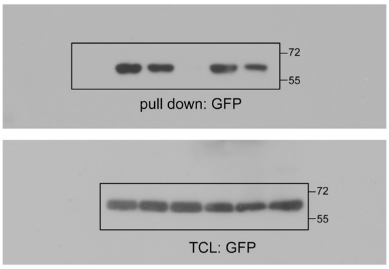

Fig. 8b

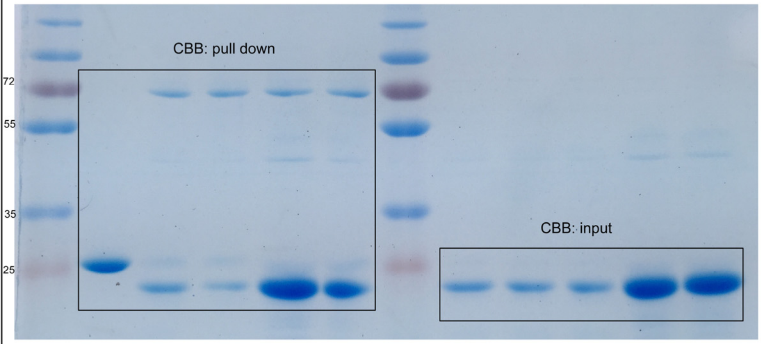

Fig. 8d

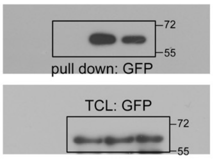

Supplementary table 1:

Primers used in the present study:

Construct name		Primer sequences
pGEX-6p-1-RAB4	F	CGGGATCCTCCGAAACCTACGATTTTTTG
	R	CCGCTCGAGCTAACAACCACACTCCTGAG
pGEX-6p-1-RAB5	F	CGGGATCCGCTAGTCGAGGCGCAACAAG
	R	CCGCTCGAGTTAGTTACTACAACACTGATTC
pGEX-6p-1-R5BD	F	GGAATTCGCTTCTATTTCTAGCCTAAAAGC
	R	CCGCTCGAGTCATGTCTCAGGAAGCTGG
pCS2-HA-PTP1B	F	GGAATTCGAGATGGAAAAGGAGTTC
	R	CGGGATCCCTATGTGTTGCTGTTGAACAGG
pXT7-Flag-VEGFR2-Cyto	F	GCTCAACTTTGGCAGATCGGTACCCACCATGGACTACAAAGACCATGACGACAAGCATATGAAGCGGGCCAATGGAGGGGAACTGAAG
	R	CTAGTGATATCAGATCTCTCGAGTTAAACAGGAGGAGAGCTCAGTG
pCMV5-Myc-VEGFR1-Cyto	F	GACTTGTTGCGGAAACATATGATAAAGACTGACTACCTATCAATTATAATGG
	R	GCCACCCGGGATCCTCTAGACTAGATGGGTGGGGTGGAGTAC
pCMV5-Myc-VEGFR2-Cyto	F	GGAATTCCATATGAAGCGGGCCAATGGAGGGGAACTG
	R	GCTCTAGATTAAACAGGAGGAGAGCTCAGTGTG
pCMV5-Myc-VEGFR3-Cyto	F	GGAATTCCATATGATCAAGACGGGCTACCTGTCC
	R	GCTCTAGATTAGTAGCTGTTGTCTGTGAAG
