# Supplementary material for: Sec14l3 potentiates VEGFR2 signaling to regulate zebrafish vasculogenesis
Source: Nat Commun. 2019 Apr 8;10:1606. doi: 10.1038/s41467-019-09604-0 (PMC6453981; doi:10.1038/s41467-019-09604-0)
Supplement: Supplementary file 3 — Description of Additional Supplementary Files [file 41467_2019_9604_MOESM3_ESM.pdf]

Description of Additional Supplementary Files

File Name: Supplementary Data 1
Description: List of differentially expressed genes between GFP+ and GFP- cells from Tg(fli1a:EGFP)y1 embryos at 22-24 hpf. The genes with log2(RPKM-GFP+ /RPKM-GFP-)>1 are shown here.

File Name: Source data
Description: Summary of raw values shown in Figure 2-7 and Supplementary Figure 4. It contains 7 sheets, each of which is named by its representative figure number.
